# Year-round efficacy of a single treatment of fluralaner injectable suspension (Bravecto Quantum^TM^) against repeated infestations with *Ixodes holocyclus* in dogs

**DOI:** 10.1186/s13071-023-05951-6

**Published:** 2023-10-20

**Authors:** Petr Fisara, Frank Guerino

**Affiliations:** 1 MSD Animal Health Australia Ltd., 26 Talavera Road, Macquarie Park, NSW 2113 Australia; 2 Merck Animal Health, 126 E. Lincoln Avenue, Rahway, NJ 07065 USA

**Keywords:** Australian paralysis tick, Bravecto, Fluralaner, Injectable, *Ixodes holocyclus*

## Abstract

**Background:**

The longer the duration of protection of an acaricide against canine infestation with *Ixodes holocyclus*, the lower the risk of gaps in tick control programs that will place dogs at risk of potentially fatal tick-induced paralysis. Two studies investigated the duration of efficacy provided by a novel injectable suspension of fluralaner (Bravecto Quantum^TM^) against this tick species.

**Methods:**

In both studies, 20 clinically healthy dogs were randomized to an untreated control group or to a group treated once, on Day 0, with the injectable fluralaner suspension (15 mg/kg). Dogs were infested with up to 25 unfed adult female *I. holocyclus* ticks on Day -1, during Weeks 1 and 2, and then at intervals no greater than approximately 3 months for the 13 months following treatment. Ticks were assessed in situ at 24 and 48 h and assessed and removed at 72 h following treatment and each subsequent infestation. Efficacy was determined by comparing arithmetic mean live tick (attached or free) counts in the treated group with the control group.

**Results:**

The untreated control dogs maintained adequate infestations for efficacy evaluations at all assessment weeks, with mean tick counts ranging from 16.2 to 21.6 in Study 1 and 14.0 to 23.5 in Study 2. The efficacy of fluralaner injectable suspension against existing infestations, determined 72 h following treatment administration, was 64.1% in Study 1 and 42.7% in Study 2. Efficacy against post-treatment infestations in Study 1 ranged from 95.7 to 100% from Week 1 through Week 57; in Study 2 efficacy was 100% at every assessment from Week 1 through Week 57. No treatment-related adverse events were recorded in either study.

**Conclusion:**

The injectable fluralaner suspension was highly effective against *I. holocyclus* infestations of dogs from one week through 13 months following a single treatment. By placing treatment with the veterinarian, killing ticks within 72 h of attachment, and providing a full year of protection, fluralaner injectable suspension can help facilitate owner compliance with tick control treatment recommendations, thus reducing the risk of canine tick paralysis.

**Graphical Abstract:**

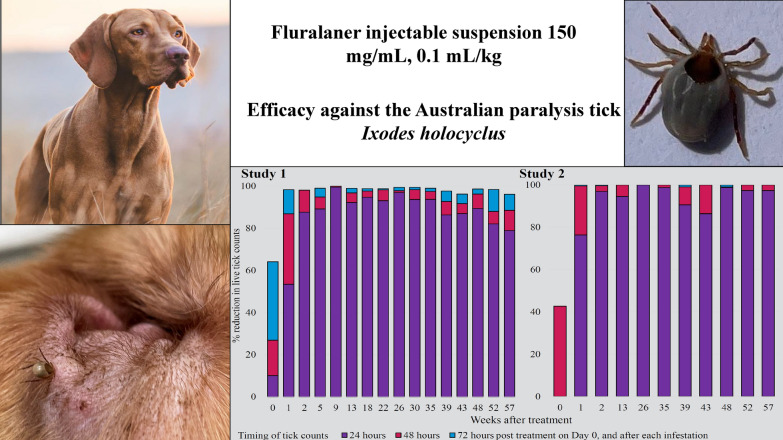

## Background

The Australian paralysis tick, *Ixodes holocyclus*, recognized since colonial times, causes an ascending flaccid paralysis in a wide range of domestic animals (including dogs, cats, horses, cattle and sheep) and humans [1]. As suggested by its alternative name, the eastern paralysis tick, the enzootic range of *I. holocyclus* extends south along the eastern coast of Australia, from northern Queensland, through New South Wales, to Gippsland in Victoria [2, 3]. Normally found within 20 km of the coast, *I. holocyclus* has been isolated in areas more than 100 km inland, including Toowoomba in Queensland and the Lower Blue Mountains in New South Wales, while epizootic cases of tick paralysis caused by *I. holocyclus* have been reported in Melbourne in the south [4, 5]. A wide range of mammalian and avian hosts facilitate completion of the *I. holocyclus* life cycle, and its typical habitat consists of bushland and wet forest areas [2, 6]. Native wildlife, particularly bandicoots, are the natural hosts of *I. holocyclus* and can carry multiple ticks without being affected, presumably due to immunity developed after exposure to the neurotoxin from an early age [7]. However, dogs and cats generally have no acquired immunity and often only one tick will cause severe illness with a potentially fatal outcome.

Extracts of salivary glands of *I. holocyclus* removed from mice after 3.5 days attachment and held for 24 h showed a marked increase in production of holocyclotoxin compared with extracts of salivary glands removed from ticks at 3 days post attachment that were held for the same amount of time [7]. Therefore, it is apparent that at some point during the fourth day after attachment there is a rapid increase in salivary gland production of holocyclotoxin, coinciding with a rapid stage of engorgement of the tick and a marked increase in the size of the salivary glands [7–9]. The risk of resulting host toxicity increases from that point as tick feeding progresses. Thus, the adult *I. holocyclus* female does not secrete clinically relevant amounts of toxin before the fourth day of attachment to the host, and the onset of paralysis is usually evident only from the fourth or beginning of the fifth day post attachment [8]. Once clinical signs begin to manifest, the paralysis may be fatal if left untreated, even if the tick(s) is removed. Therefore, to prevent the occurrence of potentially fatal paralysis in infested dogs, it is critical to kill *I. holocyclus* ticks within 3 days after attachment, prior to the increase of holocyclotoxin production in the salivary gland and the resulting onset of clinical signs.

A recent review highlights the slow progress of methods to treat and prevent tick paralysis in animals, identifying only three substantial achievements in the last 100 years of research [1]. First, the commercial availability of tick antiserum, currently the only specific anti-paralysis tick therapy available to veterinarians in Australia; second, advances in veterinary critical care have increased survival rates of even severely affected dogs and cats; third, the introduction of the isoxazoline family of compounds, credited as providing the most significant breakthrough in reducing the incidence of tick paralysis, has given dog and cat owners a reliable means of prevention.

When administered to dogs, three of the available isoxazolines, afoxolaner, lotilaner and sarolaner, require approximately monthly oral treatments to maintain efficacy against *I. holocyclus* [10]. Another isoxazoline, fluralaner, offers improved convenience to pet owners with oral and spot-on formulations, both of which provide extended periods of protection against *I. holocyclus*. A chewable tablet formulation is labeled to protect against *I. holocyclus* infestation of dogs for four months, and a spot-on formulation provides six months of protection [1, 10, 11]. This sustained fluralaner activity means fewer treatments are needed, thus reducing the risk of owner compliance failures [12]. In areas of exposure risk to *I. holocyclus*, such failures may leave a dog unprotected with potentially fatal consequences. Cases of tick paralysis are most frequently reported in spring and summer, associated with a peak in the abundance of female ticks; however, tick paralysis can occur throughout the year [13]. Therefore, a product providing year-long protection could expand on the success of the isoxazolines as strict adherence to a schedule of repeated tick control treatments throughout the year remains a challenge for dog owners [12].

An injectable fluralaner 150 mg/ml suspension has been developed for use under veterinary supervision to provide year-long protection against ticks with a single administration and thus facilitate owner compliance with tick control treatment recommendations. Two laboratory studies, using similar protocols, investigated the efficacy of a single treatment with this fluralaner formulation against canine infestations with *I. holocyclus* ticks for at least 13 months (398 days, approximately 57 weeks).

## Methods

Each two-arm (untreated control, fluralaner-treated) study was randomized, non-masked and complete block design, conducted in alignment with VICH GCP guidelines [International Co-operation on Harmonisation of Technical Requirements for Registration of Veterinary Medicinal Products, Good Clinical Practice (GL9)], June 2000 [14]; The World Association for the Advancement of Veterinary Parasitology (WAAVP) second edition: guidelines for evaluating the efficacy of parasiticides for the treatment, prevention and control of flea and tick infestation on dogs and cats [15]; and the Australian Pesticides and Veterinary Medicines Authority (APVMA) Preamble for the WAAVP guideline for fleas and ticks on dogs and cats [16]. The study was a Small Scale Trial as defined by the APVMA. For Study 1, Animal Ethics Committee approval was obtained prior to the commencement of the study from the Wongaburra Research Centre Animal Ethics Committee. An Animal Research Authority and a Certificate of Approval to provide ethics clearance were obtained for Study 2 from the New South Wales Department of Primary Industries Secretary’s Animal Care and Ethics Committee.

Guidelines recommend that in studies to investigate acaricidal efficacy, at least six dogs should be included in each study group, with each dog representing an experimental unit [15]. To ensure infestations and efficacy assessments were completed on a sufficient number of dogs, 10 were included in each group. Application of ticks to predefined locations, recording and identification and careful examination of those locations by non-masked study personnel ensured that accurate tick counts were completed at each assessment.

### Dogs and management

In both studies, 20 clinically healthy dogs, uniquely identified by name and microchip number, were selected from a group of 24 dogs based on tick-carrying capacity that had been determined by infestations and assessments in the week prior to treatment. For inclusion, dogs had to be at least six months of age on the day of treatment (Day 0), could not have been treated with any long-acting insecticide or acaricide within the previous 90 days, nor fluralaner within the previous 180 days. Dogs were also required to have an appropriate temperament to allow completion of study procedures.

Whilst carrying ticks, dogs were housed individually such that no physical contact was possible between dogs. On days when the dogs were not infested with ticks, they were housed individually or in socially compatible pairs or groups of three in adjacent pens. To prevent any cross-group contact, treatment groups were separated inside the kennels either by a central corridor with a solid partition running along the center or by pens housing non-study dogs. The dogs were regularly allowed access to separate dedicated exercise areas designed to prevent any potential exposure of control dogs to fluralaner-treated dogs. Feed was a standard commercial dry canine diet provided at least once daily, and fresh water was provided ad libitum in bowls or automatic troughs. In Study 1, dogs were either Foxhound or Foxhound cross, aged between four and 10 years, with a weight range of 31.9 to 42.0 kg. The 20 dogs selected in Study 2, comprised of pure and mixed-breed Beagles and a Foxhound, were aged between 10 months and 7 years.

Dogs in Study 1 were hyperimmunised against holocyclotoxin in the weeks leading up to the study by repeated infestations with low numbers of *I. holocyclus* ticks. The immunity against the effect of the tick toxin was maintained in the untreated group dogs throughout the study by low level tick infestations during the intervals between the infestations for efficacy evaluations. Dogs in Study 2 were naïve and therefore were subjected to tick paralysis challenge assessments between three and 10 days after each infestation (i.e., after all ticks had been removed at 72 h). The assessments consisted of various types of exercises (for example jumping onto a small platform or elevated bed, etc.) that each dog had to complete daily. Observations of any lack of coordination or hind limb weakness would indicate early onset of tick paralysis and allow appropriate therapeutic measures to be taken. Two untreated dogs in Study 1 that fully recovered from signs of mild post-infestation tick paralysis were able to continue and complete the study. No dogs developed signs of paralysis in Study 2.

### Tick infestations and assessments

The *I. holocyclus* ticks for Study 1 were collected from localities of the Northern Rivers region of New South Wales, South East Queensland and Far Northern Queensland. The ticks were stored in jars and kept in an incubator at approximately 12 to 14 °C with high humidity. Study 2 ticks were collected from localities of the Northern Rivers region of New South Wales and stored in glass jars under similar conditions (temperature approximately 16 °C with high humidity).

For tick challenges, dogs were either sedated, removed from their pens and taken to the infestation room where adult unfed female *I. holocyclus* ticks were manually applied to multiple sites (Study 2) or were infested in their kennels without sedation using a similar infestation method (Study 1). Anatomical infestation sites were located according to the tick’s natural predilections, on the head, ears, shoulders and dorsal midline. Each application site was accurately documented to facilitate finding ticks during efficacy assessments.

To determine the tick-carrying capacity of each dog, and to provide tick count data for randomization, pretreatment infestations were performed on Days -7 or -6. All dogs in both studies were again infested on Day -1 and in Weeks 1, 2, 13, 26, 35, 39, 43, 48, 52 and 57. In Study 1, infestations were also completed in Weeks 5, 9, 18, 22 and 30, but not in Study 2 due to a shortage of ticks. All infestations were with 25 unfed, adult female *I. holocyclus*, except in Study 2 in Week 13 (15 ticks per dog) and Week 26 (23 ticks per dog) when fewer ticks were available. To limit local reactions to tick attachment sites, antihistamines were allowed to be administered on days of infestations and up to 24 h after tick removal.

The pretreatment tick assessments were conducted on Day -4 or -3 (72 h post infestation). Tick counts for determination of product efficacy against existing infestations at the time of treatment were performed on each dog, at 24 and 48 h (Study 2) and 24, 48 and 72 h (Study 1) post treatment (each ± 4 h). Efficacy against post-treatment infestations was evaluated in both studies at 24, 48 and 72 h following each infestation. The 24- and 48-h assessments were completed without removing ticks. All ticks were removed after the 72-h assessment. Each tick was classified as live (attached or free) or dead (attached or free). All ticks classified as dead were removed and stimulated by assessor breath and by manual probing to confirm their dead status. If any tick(s) could not be accounted for, the dog was thoroughly searched by visual inspection and digital palpation. The total live tick counts recorded at the 72 h post-infestation assessment were used to determine efficacy in each assessment week.

### Treatment groups

The 20 dogs with the highest live tick counts at 72 h post infestation on Days -7 or -6 were ranked by count and blocked into pairs. Within pairs, each dog was randomly allocated to a treatment group using the *randbetween* function in Microsoft Excel, resulting in 10 dogs per treatment group. Dogs allocated to the control group did not receive any treatment, while dogs assigned to the fluralaner-treated group received a single subcutaneous injection of the fluralaner suspension (150 mg/ml), administered on a single occasion (Day 0) in the dorsoscapular region. The dogs were treated at the fluralaner label dose rate of 15 mg/kg (0.1 ml/kg), calculated using body weights recorded on Days -3 or -1. A 5-min observation period immediately following treatment was carried out for each dog for signs of adverse reactions. The dogs were also observed for general health at 24 and 48 h post treatment. Injection site assessments were conducted pre-treatment on Day 0 and on Days 1, 2, 3, 4, 7, 10 and 14. On each occasion, the observations were compared with the baseline pretreatment observations of each dog. The assessments included palpation to determine the presence of any swelling, erythema, heat or pain. General health observations of each dog were completed at least once a day for the duration of each study.

### Statistical methods

The primary endpoint was efficacy at 48 h (Study 2) and 72 h (Study 1) post treatment and at 72 h following each subsequent infestation. Live tick count data were transformed using the *y* = log_e_(*x* + 1) transformation and analyzed using a linear mixed-effect model that included treatment group as a fixed effect and block as a random effect. Separate analyses were conducted for each time point. A Kenward-Rogers adjustment was used to determine the denominator degree of freedom for hypothesis testing. At each time point, a two-sided t-test was used to assess whether the difference in least-squares means between the fluralaner and control groups was statistically significant at *P* ≤ 0.05. These comparisons were performed on the transformed scale. The least-squares means of tick counts were then back-transformed to obtain the geometric mean tick counts on the response scale. Data for live tick counts were summarized as arithmetic and geometric means by treatment group and time point. Efficacy was determined as the percent reduction in arithmetic and geometric means in the treated group compared with the control group using the formula:

$$\text{\% reduction}=100 \times \frac{\text{mean\,count\,control\,group}-\text{mean\,count\,treated\,group}}{\text{mean\,count\,control\,group}}$$

R version 4.0.5 was the software used for analysis [17].

## Results and discussion

The untreated control dogs maintained an adequate level of infestations at all assessment time points throughout both studies, with arithmetic mean tick counts ranging in Study 1 from 16.2 to 21.7 and in Study 2 from 14.0 to 23.5 (Tables 1 and 2). The efficacy against existing infestations was respectively 64.1% and 72.9% by arithmetic and geometric means in Study 1 and 42.7% and 64.2% in Study 2. The efficacy following subsequent post-treatment tick infestations ranged in Study 1 from 95.7 to 100% and in Study 2 was 100% at all 72-h assessments from Week 1 through 13 months post treatment. Based on the significant reduction (*P* ≤ 0.05) in live tick counts compared to the untreated control group and ≥ 95.7% efficacy based on arithmetic and geometric means, in both studies the fluralaner 150 mg/ml injectable suspension was effective in controlling *I. holocyclus* tick infestations from Week 1 through 57 (13 months) following administration.

**Table 1:** Study 1, arithmetic (geometric) mean counts of live *Ixodes holocyclus* and efficacy at 72 h post treatment and after each infestation following a single treatment of dogs with fluralaner subcutaneous injection

Time after treatment	Mean live tick counts^a^ arithmetic (geometric)		% efficacy, arithmetic (geometric) mean	Statistics	
	Control	Fluralaner		*t*-value	*P*-value
3 days (Week 0)	19.8 (19.6)	7.1 (5.3)	64.1 (72.9)	*t*_*9*_ = 4.7	0.001
Week 1	17.7 (17.3)	0.3 (0.2)	98.3 (98.7)	*t*_*9*_ = 26.0	< 0.001
Week 2	21.2 (21.0)	0.4 (0.2)	98.1 (98.8)	*t*_*9*_ = 20.3	< 0.001
Week 5	21.0 (20.5)	0.2 (0.1)	99.0 (99.4)	*t*_*9*_ = 22.9	< 0.001
Week 9	16.2 (15.5)	0.0 (0.0)	100.0 (100.0)	*t*_*9*_ = 27.2	< 0.001
Week 13	18.5 (17.9)	0.2 (0.1)	98.9 (99.2)	*t*_*9*_ = 24.4	< 0.001
Week 18	16.8 (16.1)	0.2 (0.1)	98.8 (99.1)	*t*_*9*_ = 24.3	< 0.001
Week 22	16.4 (16.0)	0.2 (0.1)	98.8 (99.3)	*t*_*9*_ = 22.7	< 0.001
Week 26	17.3 (16.7)	0.1 (0.1)	99.4 (99.6)	*t*_*9*_ = 27.9	< 0.001
Week 30	17.8 (17.4)	0.1 (0.1)	99.4 (99.6)	*t*_*8*_ = 34.1	< 0.001
Week 35	19.4 (19.2)	0.2 (0.1)	99.0 (99.2)	*t*_*9*_ = 32.3	< 0.001
Week 39	16.4 (16.2)	0.4 (0.3)	97.6 (98.3)	*t*_*9*_ = 18.5	< 0.001
Week 43	18.8 (18.5)	0.8 (0.5)	95.7 (97.3)	*t*_*9*_ = 13.1	< 0.001
Week 48	21.6 (21.5)	0.3 (0.2)	98.6 (99.1)	*t*_*9*_ = 22.8	< 0.001
Week 52	18.6 (18.1)	0.3 (0.2)	98.4 (98.7)	*t*_*9*_ = 20.8	< 0.001
Week 57	18.1 (17.9)	0.7 (0.5)	96.1 (97.4)	*t*_*9*_ = 14.5	< 0.001

^a^Dogs were infested with 25 ticks at all infestations

**Table 2:** Study 2, arithmetic (geometric) mean counts of live *Ixodes holocyclus* and efficacy at 72 h post treatment and after each infestation following a single treatment of dogs with fluralaner subcutaneous injection

Time after treatment	Mean live tick counts^a^ arithmetic (geometric)		% efficacy, arithmetic (geometric) mean	Statistics	
	Control	Fluralaner		*t-*value	*P*-value
2 days (Week 0)	22.5 (22.4)	12.9 (8.0)	42.7 (64.2)	*t*_*9*_ = 2.4	0.038
Week 1	17.7 (17.1)	0.0 (0.0)	100.0 (100.0)	*t*_*9*_ = 33.1	< 0.001
Week 2	21.3 (21.1)	0.0 (0.0)	100.0 (100.0)	*t*_*9*_ = 71.9	< 0.001
Week 13	14.0 (14.0)	0.0 (0.0)	100.0 (100.0)	*t*_*9*_ = 132.2	< 0.001
Week 26	20.2 (20.0)	0.0 (0.0)	100.0 (100.0)	*t*_*9*_ = 71.3	< 0.001
Week 35	23.5 (23.4)	0.0 (0.0)	100.0 (100.0)	*t*_*9*_ = 147.1	< 0.001
Week 39	22.6 (22.5)	0.0 (0.0)	100.0 (100.0)	*t*_*9*_ = 102.9	< 0.001
Week 43	22.8 (22.7)	0.0 (0.0)	100.0 (100.0)	*t*_*9*_ = 140.9	< 0.001
Week 48	22.7 (22.6)	0.0 (0.0)	100.0 (100.0)	*t*_*9*_ = 114.8	< 0.001
Week 52	22.1 (22.0)	0.0 (0.0)	100.0 (100.0)	*t*_*9*_ = 104.3	< 0.001
Week 57	21.8 (21.6)	0.0 (0.0)	100.0 (100.0)	*t*_*9*_ = 66.7	< 0.001

^a^Dogs were infested with 25 ticks except for Weeks 13 and 26 when they were infested with 15 and 23 ticks respectively due to a tick shortage

**Fig. 1:**
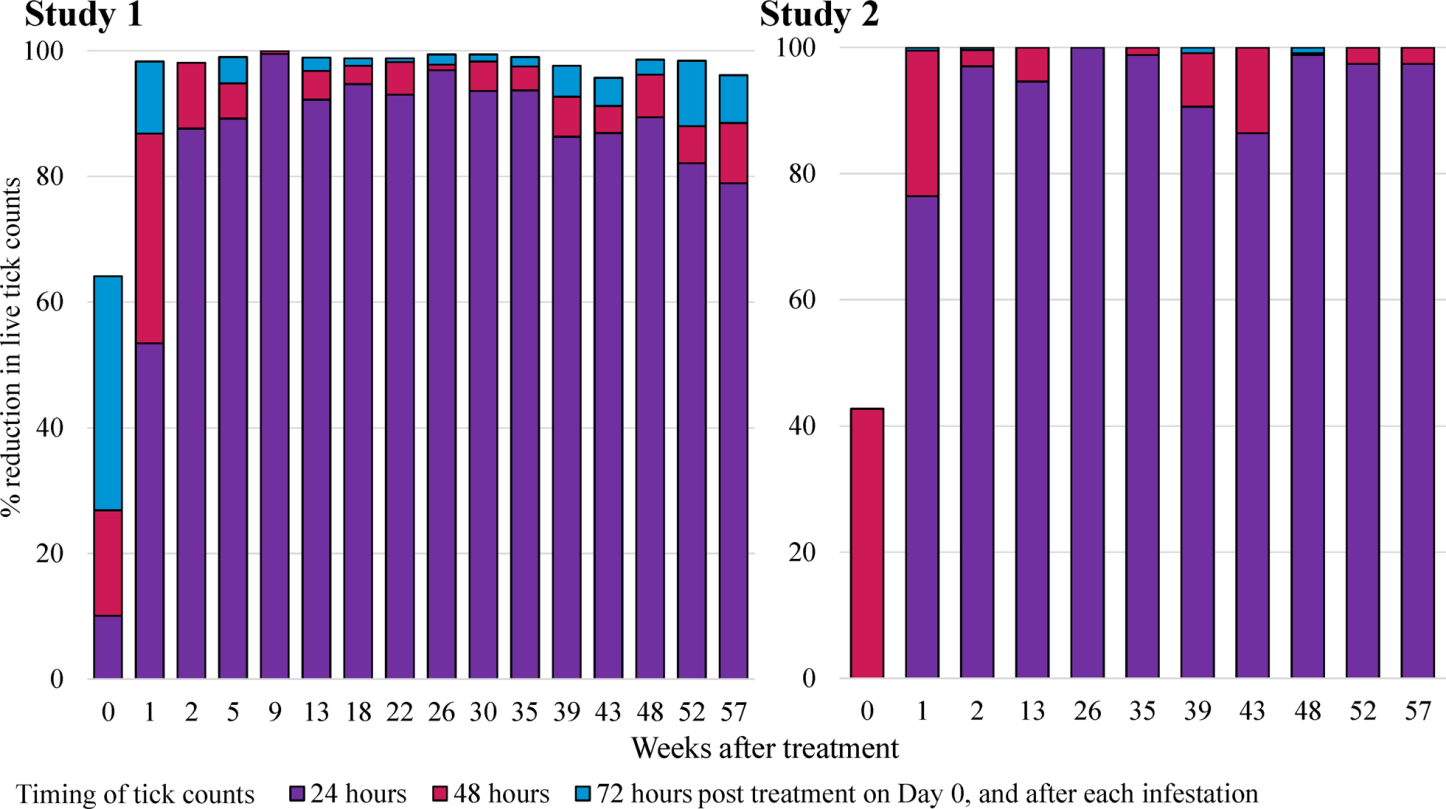
Percentage reduction in live *Ixodes holocyclus* counts in dogs treated with fluralaner injectable suspension on Day 0 compared with untreated controls at 24 and 48 h (Study 2) and 24, 48 and 72 h (Study 1) post treatment and at 24, 48 and 72 h following each infestation in both studies

The in situ post-treatment assessments demonstrated a high level of efficacy against *I. holocyclus* at the 24- and 48-h tick counts following each post-treatment infestation. In treated dogs, the mortality of *I. holocyclus*, relative to the untreated control group, exceeded 90% at 24 h post infestation between Weeks 13 and 35 in Study 1 and Weeks 2 through 57 in Study 2 (Fig. 1). At 48 h post infestation, tick mortality in Study 1 was > 90% from Week 2 through Week 48 and in Study 2 was > 99% from Week 1 through Week 57.

No treatment-related adverse events were recorded in either of the two studies. In Study 1, diarrhea was observed in two control group dogs and five fluralaner-treated dogs between 6 and 13 months after treatment. These events were linked to an increased incidence of diarrhea in the general dog population at the facility, attributed most likely to endemic hookworm infections and overgrowth of *Clostridium perfringens*. In Study 1, a dog in the untreated control group was  euthanized during Week 27 because of a metastatic hepatic neoplasia. A replacement dog was introduced from the Week 35 infestation onwards, leaving nine control group dogs for the Week 30 infestation. A mild tick paralysis of that dog resulted in its exclusion from the Week 48 infestation.

Effective blood levels following treatment with fluralaner injectable suspension are reached after 3 days [18]. Therefore, it is recommended dogs at risk of exposure to *I. holocyclus* should be thoroughly searched daily and any live ticks removed during this period. Dogs should also be searched just prior to treatment as existing ticks will not be killed immediately.

## Conclusion

The injectable fluralaner suspension was highly effective against *I. holocyclus* infestations of dogs from one week through 13 months following a single treatment. By placing treatment with the veterinarian, killing ticks within 72 h of attachment and providing a full year of protection, fluralaner injectable suspension can help facilitate owner compliance with tick control treatment recommendations thus reducing the risk of canine tick paralysis. Regardless of the consistently high efficacy seen in this study, no product can be 100% effective in all animals, and vigilance against this tick remains important.

## Data Availability

Data from this study are proprietary and maintained by Merck Animal Health, Rahway, NJ, USA.

## Abbreviations

APVMA: Australian Pesticides and Veterinary Medicines Authority
GCP: Good Clinical Practice
VICH: International Co-operation on Harmonisation of Technical Requirements for Registration of Veterinary Medicinal Products
WAAVP: World Association for the Advancement of Veterinary Parasitology
